# Reporting phenotypes in mouse models when considering body size as a potential confounder

**DOI:** 10.1186/s13326-016-0050-8

**Published:** 2016-02-09

**Authors:** Anika Oellrich, Terrence F. Meehan, Helen Parkinson, Sirarat Sarntivijai, Jacqueline K. White, Natasha A. Karp

**Affiliations:** Mouse Informatics Group, Wellcome Trust Sanger Institute, Hinxton, Cambridgeshire UK; Social Genetic & Developmental Psychiatry, King’s College London, London, UK; Samples, Phenotypes and Ontologies, European Molecular Biology Laboratory—European Bioinformatics Institute, Hinxton, Cambridge UK; Samples, Phenotypes and Ontologies, European Bioinformatics Institute (EMBL-EBI), European Molecular Biology Laboratory, Wellcome Trust Genome Campus, Hinxton, Cambridge CB10 1SD UK; The Centre for Therapeutic Target Validation, Wellcome Trust Genome Campus, Hinxton, Cambridge CB10 1SD UK; Mouse Genetics Project, Wellcome Trust Sanger Institute, Hinxton, Cambridgeshire UK

## Abstract

Genotype-phenotype studies aim to identify causative relationships between genes and phenotypes. The International Mouse Phenotyping Consortium is a high throughput phenotyping program whose goal is to collect phenotype data for a knockout mouse strain of every protein coding gene. The scale of the project requires an automatic analysis pipeline to detect abnormal phenotypes, and disseminate the resulting gene-phenotype annotation data into public resources. A body weight phenotype is a common result of knockout studies. As body weight correlates with many other biological traits, this challenges the interpretation of related gene-phenotype associations. Co-correlation can lead to gene-phenotype associations that are potentially misleading. Here we use statistical modelling to account for body weight as a potential confounder to assess the impact. We find that there is a considerable impact on previously established gene-phenotype associations due to an increase in sensitivity as well as the confounding effect. We investigated the existing ontologies to represent this phenotypic information and we explored ways to ontologically represent the results of the influence of confounders on gene-phenotype associations. With the scale of data being disseminated within the high throughput programs and the range of downstream studies that utilise these data, it is critical to consider how we improve the quality of the disseminated data and provide a robust ontological representation.

**Electronic supplementary material:**

The online version of this article (doi:10.1186/s13326-016-0050-8) contains supplementary material, which is available to authorized users.

## Introduction

**Fig. 1:**
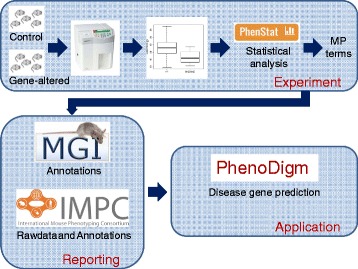
The phenotyping pipeline. The high throughput phenotyping pipeline integrates a series of screens to assess the impact of the genotype amendment on a variety of disease-related and biological systems. Statistical analysis comparing data from the gene altered and control animals allows the identification of abnormal phenotypes, assignment of ontology annotation and dissemination of data to public database for data mining across species and studies. IMPC represents the International Mouse Phenotyping Consortium web portal [26] where the data is collected, analysed and annotations disseminated. Annotations are assigned using the Mammalian phenotype ontology (MP)

In genotype-phenotype studies, one approach to identify abnormal phenotypes is a statistical comparison of data collected from control and gene-altered animals. In this paper we use the International Mouse Phenotyping Consortium (IMPC) statistical analysis pipeline as a use case study [1]. The goal of the IMPC is to produce and phenotypically characterise 20,000 knockout mouse strains in a reproducible manner across multiple research centres. This high-throughput phenotyping is based on a pipeline concept where a mouse is characterised in a series of phenotype screens underpinned by standard operating procedures defined by the IMPC in the International Mouse Phenotyping Resource of Standardised Screens (IMPReSS) resource [2]. This pipeline approach characterises seven males and seven females for each knockout line and results in data for over 200 physiological variables that cover a variety of disease-related and biological systems. As the scale of the program requires the statistical analysis to be automated, we have developed the statistical package PhenStat [3] to analyse genotype-phenotype associations. In order to provide a consistent representation of results, area experts have reviewed the IMPReSS screens and have associated one or more terms from the Mammalian Phenotype Ontology (MP) [4] with each variable. For example, the variable “fasted blood glucose concentration” is associated to three MP terms: “abnormal-”, “increased-”, and “decreased-” “-fasted circulating glucose level”. Using this approach, abnormal phenotypes identified via statistical analysis are summarised as gene-phenotype associations, easily understood by the biological community and facilitating dissemination to the community (Fig. 1). The current analysis pipeline only takes sex into consideration when identifying abnormal phenotypes. Sharing these gene-phenotype annotations also enables data mining across species and studies e.g. for disease gene candidate discovery, pharmacogenetics and evolutionary studies [5–7].

During the statistical comparison of control and gene-altered data, confounding variables associated with both the genotype change and the phenotype of interest can lead to an association that is true but potentially biologically misleading. The presence of the confounding relationship can lead to errors in the estimates of the relationship between the treatment of interest (here the genotype change) and the variable of interest (here the phenotype). Good experimental design can manage many potential confounders using standardisation e.g. with the potential confounder of age, the study would only test animals of the same age. An alternative strategy is randomisation, in which animals of multiple ages are tested in both control and the experimental knockout group. Yet another strategy is grouping (blocking) according to a confounding variable (e.g. pup or adult). Depending on the strategy applied, the final annotation could be specific to one particular age. To minimise the potential impact of confounders within IMPC, the community identified critical sources of variation in screens and used this to develop a standardised operating procedure which, where possible, minimises variation and captures potential sources of variation as metadata with each dataset. Metadata parameters (e.g. X-ray equipment) are included in the IMPReSS protocols and submitted metadata is used to determine comparison groups as part of the statistical analysis pipeline.

In many research studies, it is not possible to manage confounding variables during the design. For example, in many gene knockout studies, the knockout animals show an abnormal body weight change. Therefore, any other phenotypic traits (e.g. abnormal body fat mass MP:0012320) that correlate with body weight will also be impacted. As the experimenter cannot control this potential confounder through the design, it is necessary to consider statistical methods for non-equivalent groups [8]. These include regression methods where the confounder is treated as a covariate, meaning the statistical test will assess the effect of the genotype on the phenotype after adjusting for the confounder’s relationship. This requires a dataset to be processed twice, first without and then with the confounder in the statistical analysis; giving two sets of results for the test of genotype. This granularity has a high potential value to improve our interpretation of the relationship between a gene and associated phenotypes. However, the vast majority of MP terms represent absolute phenotype changes in a variable of interest. The Mouse Genome Informatics database (MGI) [9] developed MP to manually curate the scientific literature. However, only in rare, clear cause and effect cases are confounding variables represented as part of the ontology. For example, the term “progressive muscle weakness” (MP:0000748) is defined as a muscle weakness that increases with time. Time or age are clearly contributing to the severity of the phenotype and thus represent knowledge that should be represented in the ontology [10]. However, in many studies a confounding variable is noted by authors’ to contribute to a phenotype, but a clear cause and effect relationship is not established. The current mechanism employed by MGI is to manage confounders at the level of annotation by utilising free text qualifiers. For example, the curator will note if an author states body weight was a confounder when associating a phenotype to a genotype. With the scale of IMPC data and the automated aspect of statistical analysis and subsequent annotation, we have the potential to manage these issues in a consistent way and through standardisation better support downstream informatic analysis. The interest in including body weight as a covariate, in both high throughput phenotyping studies and small scale studies, is growing [8, 11–13]. This manuscript aims to raise awareness of the issues and demonstrate the potential value of addressing the problems. We then identify adaptations to the existing mechanisms utilised by the community that could address this new aspect where we wish to disseminate the outcome of an analysis that considers body weight as a confounder.

Data and scripts used to investigate and demonstrate issues presented within this manuscript are available at Zenodo [14].

### Body weight as a confounder

Body weight is a highly heritable trait and is estimated to be a potential latent variable in a third of experiments studying knockout mice [11]. It has been shown that body weight correlates with many variables, ranging from body composition to clinical chemistry [15]. Including body weight in the computational analysis allows the phenotype to be assessed after adjusting for weight differences (see Additional file 1: Supplementary Methods).

Dual analysis can lead to annotations that differ depending on the analysis pipeline (Table 1) as one can then assess whether the phenotype has changed in a relative and absolute sense. For example, when the abnormality is due solely to correlation with a body weight phenotype, then the inclusion of body weight as a covariate adjusts for this confounding relationship and the phenotype (as a relative term) would no longer be called significant (Table 1 row 1). Alternatively, a line may only have a significant abnormal annotation in the analysis pipeline when body weight is included. The inclusion of body weight accounts for more variation in the data, increasing the sensitivity to detect other phenotypes (Table 1, row 3). Lines can also be significant in both analysis pipelines (Table 1, row 4), and this can arise from two scenarios which differ in whether there is a body weight difference or not. As the difference arises from presence or absence of a body weight difference, it could be argued that the interpretation could be driven by the assessment of whether a body weight phenotype was also annotated. However, a body weight phenotype might be the reason statistically, but the abnormal body weight annotation might not have been made due to low statistical sensitivity (ability to detect a difference).

**Table 1:** Possible outcomes of a dual analysis process

Row	A1	A2: + weight	Conclusion	Insight
1	+	-	Absolute phenotype ∆	No longer significant–confounded by BW
2	-	-	No abnormality	
3	-	+	Relative phenotype ∆	Adding weight increases sensitivity to detect ∆
4	+	+	Absolute phenotype ∆ and relative phenotype ∆	Two scenarios
				1. BW difference: still there is a significant ∆ as ∆ larger/smaller than expect for BW difference
				2. BW same: a significant ∆. Weight explains variation but does not lead to phenotype difference.

Possible outcomes when assessing for a genotype effect for a variable of interest when the analysis excludes (A1) or includes body weight as a covariate (A2). In this table, + indicates a statistically significant genotype effect;−indicates a non-significant genotype effect; ∆ indicates change; BW indicates body weight

**Fig. 2:**
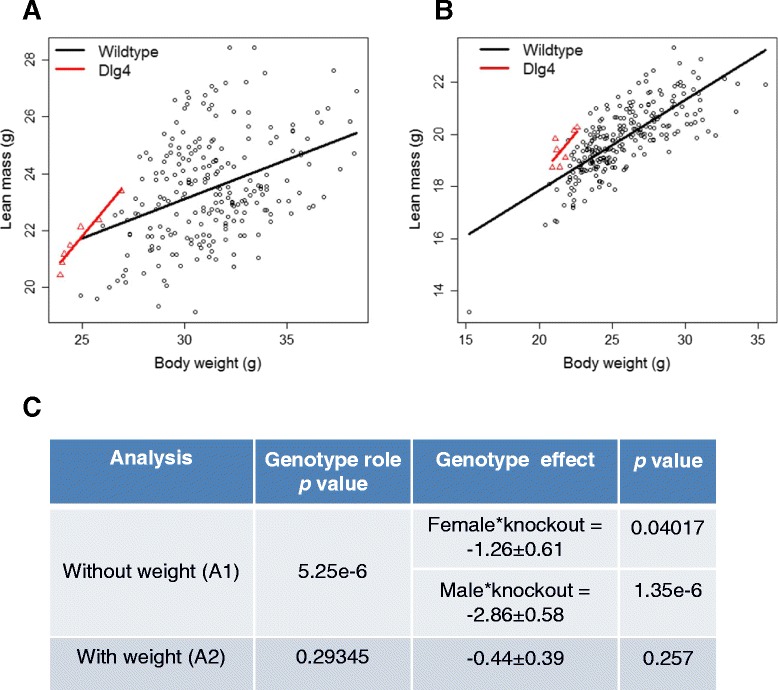
Example line *Dlg4*, where body weight confounds the phenotype. Body composition data were collected with a dual-energy X-ray absorptiometry at 14 weeks of age for the *Dlg4* ^tm1e (*EUCOMM*) *Wtsi*^/*Dlg4* ^*tm1e* (*EUCOMM*) *Wtsi*^ knockout line on the C57BL6/N genetic background. The comparison was based on 249 female and 227 male wildtype mice and 7 female and 7 male knockout mice. **a** A scatterplot of the lean mass readings for the control and knockout animals for the males. **b** A scatterplot of the lean mass readings for the control and knockout animals for the females. **c** The genotype estimate with associated standard error and statistical significance when estimated using standard methodology (A1: Analysis Pipeline 1) and then after inclusion of body weight as a covariate (A2: Analysis Pipeline 2). As there was evidence of sexual dimorphism in the phenotype in A1, the genotype effect was estimated for male and female knockout mice separately. The scatter plots and analysis highlight how a body weight phenotype is observed in both sexes of the knockout animals and as the lean mass is associated with body weight, a statistically significant difference is seen in the lean mass until assessed as a relative abnormality

**Fig. 3:**
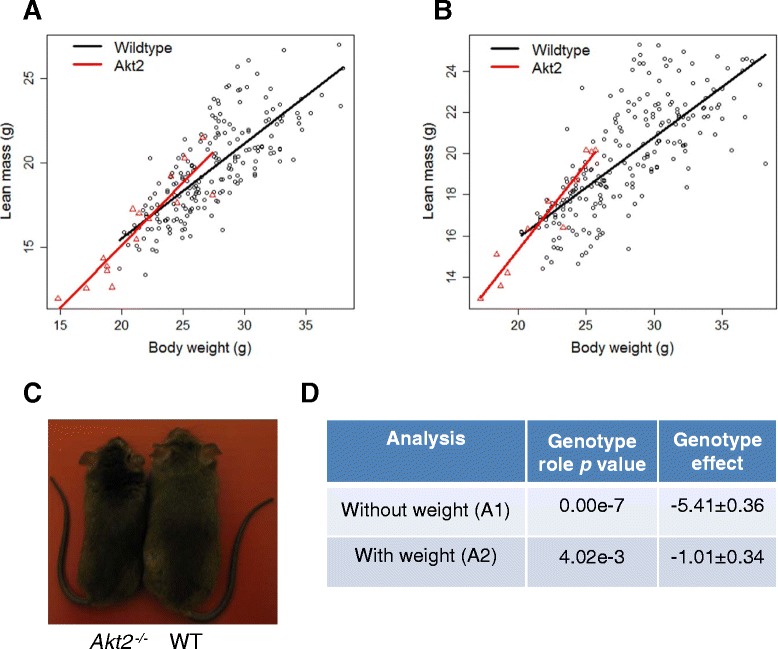
Example line *Akt2*, where body weight confuses the phenotype interpretation. Body composition data were collected with dual-energy X-ray absorptiometry at 14 weeks of age for the *Akt2* ^*tm1e* (*KOMP*) *Wtsi*^/*Akt2* ^*tm1e* (*KOMP*) *Wtsi*^ knockout line on the 129S5/SvEv^Brd/Wtsi^;129S7/SvEv^Brd/Wtsi^ genetic background. The comparison was based on 71 female and 84 male wildtype mice and 12 female and 14 male knockout mice. **a** A scatterplot of the lean mass readings for the wildtype and knockout animals for the males. **b** A scatterplot of the lean mass readings for the wildtype and knockout animals for the females. **c** Representative photograph demonstrating body weight phenotype. **d** The genotype estimate with associated standard error and statistical significance when estimated using the standard methodology (A1: Analysis Pipeline 1) and then after inclusion of body weight as a covariate (A2: Analysis Pipeline 2). The scatterplots of the lean mass against body weight highlight that there is a clear body weight phenotype and the difference between the knockouts and wildtype mice cannot be fully explained by the association between lean mass and body weight

For example, consider the *Dlg4* knockout mouse line that has a reduced body weight phenotype (MP:0001262) where we are also interested in assessing the impact of the genotype change on body composition. As body composition variables such as lean mass (MP:00039590) are dependent on the body weight, we would expect these to be decreased as an *absolute* phenotype change (Fig. 2a and b). When we include body weight in the analysis, we find that the change in lean mass is as expected for the change in body weight and determine that the phenotype *relative* to body weight is not statistically significant (Fig. 2c) (Equivalent to row 1 of Table 1). The knockout gene *Akt2* similarly has a body weight phenotype (Fig. 3a). However, the inclusion of body weight in the analysis finds that the relative lean mass is still statistically significant (Fig. 3b-d) (Equivalent to row 4 of Table 1). By adding a statistical step where we study the phenotype after adjusting for body weight, we gain a more detailed understanding of the impact of the genotype on the phenotype.

Even in cases where it is clear that body weight is truly acting as a confounding variable and is not just explaining data variance (Table 1, row 1), causality is not determined. For example, we cannot assess whether the lean mass is lower in the *Dlg4* line because the body weight is fundamentally lower or because there is less lean mass leading to a lower body weight. The refinement is therefore to consider the data and assess for both relative and absolute changes and disseminate this richness.

### Magnitude of impact and complexity

**Fig. 4:**
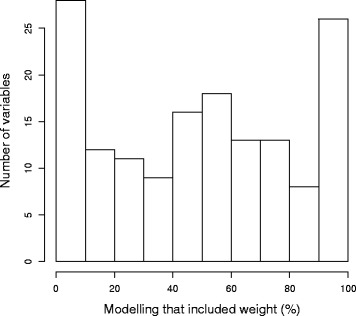
The inclusion of weight as a source of variation. The distribution of weight inclusion in the PhenStat analysis of 85086 control-knockout datasets which covers 154 variables (average number datasets = 552) from the high throughput phenotyping data collected at the WTSI MGP. The PhenStat analysis was completed using the Mixed Model framework with a starting model that included weight. The model optimisation process means that the final model will only include weight if it is statistically significant in explaining variation in the data (*p* < 0.05)

**Fig. 5:**
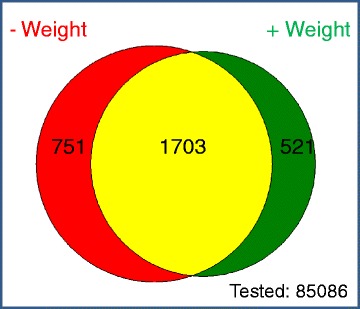
The impact of including body weight as a covariate on abnormal phenotype annotations. The relationship between the abnormal phenotype annotations made when assessing for a genotype effect by processing through A1 (standard statistical analysis pipeline) and A2 (statistical analysis including body weight as a covariate). The analysis used a mixed model method implemented within PhenStat [9] on data collected by the WTSI MGP (for more details see Additional file 1: Supplementary Methods). Shown in red are those annotations, where the phenotype difference was due to the confounding effect of body weight (row 1 of Table 1). Shown in green are those annotations where adding weight to the analysis has increased sensitivity (row 3 of Table 1). Shown in yellow are annotations made in common by both pipelines (row 4 of Table 1). Data available from Zenodo [14]

The Wellcome Trust Sanger Institute’s (WTSI) Mouse Genetics Project (MGP) is part of the IMPC community effort to phenotype knockouts for all mouse protein coding genes [16]. To support the argument that we need to consider body weight, we provide the results of a supporting analysis of the WTSI MGP data (see Additional file 1: Supplementary Methods for details). Firstly, we demonstrate that for the majority of the dataset, weight is often a significant source of variation (Fig. 4). This is seen across biological processes and not only includes screens that assess body composition but also screens such as plasma chemistry. Secondly, this data allows us to compare the impact of the dual analysis process using the standard pipeline (A1) which does not account for weight, compared to the additional analysis pipeline (A2) including body weight as a covariate. This analysis demonstrates that including body weight has a significant impact on the final abnormality annotations (Fig. 5). We find that 70 % of the abnormal annotations from the standard pipeline were also annotated when we included body weight in the analysis. Furthermore, we find that 30 % of annotations in the standard pipeline (A1) were no longer significant in A2 as they arose from the confounding impact of body weight (equivalent to row 1 of Table 1). 21 % of the annotations in A2 only occurred when body weight was included and arose from the increase in sensitivity from including body weight (equivalent to row 3 of Table 1).

### Challenges applying existing solutions

As demonstrated with the provided analysis, taking confounding variables such as body weight into account may lead to a more comprehensive dataset and should be further investigated (see Table 1). The dissemination of the resulting annotation data is achieved through a collaboration between different communities. IMPC currently uses MP to annotate genes with phenotypes. MP is a pre-composed phenotype ontology in which every concept semantically describes one particular phenotype, e.g. decreased lean body mass (MP:0003961). While this paper generalises to gene-phenotype annotations, MGI distinguishes further the additional data such as the genetic background or the sex if there is a difference between male and female mice. While the majority of the annotations contained in MGI do not take confounders into consideration, sex in the presence of sexual dimorphism could be regarded as such and is captured at times in MGI. For example, the gene *Dmxl2* [17] exhibits sexual dimorphism such that the phenotype was only found to be significant in the females of heterozygous mice and this is recorded as a curator note.

Body weight is not the only variable that could be used to adjust for the size of the animal; alternatives include body length or width. Adjustment for body size as a confounder has unique challenges (see section ‘**Body weight as a confounder**’) and particular issues with determining causality. Thus, we investigated solutions for the standardised reporting of phenotypes after considering body weight as a confounder as a relative phenotype change within existing semantic frameworks and report our findings here. Potential solutions were limited to those we believed could be implemented as they had the lowest modification requirements on the existing dissemination pipelines, such as those maintained by MGI. We note that the discussed solutions only focus on future dissemination but do not include strategies on how to deal with legacy data.

### Use of pre-composed ontologies

As mentioned before, the vast majority of phenotypes represented in the current version of MP constitute absolute changes that cannot readily be applied to confounder-adjusted phenotypes. In order to represent the results of a confounder-sensitive analysis, additional MP concepts would be needed that would allow a user to report relative phenotype changes (see column 2, Table 1, rows 3 and 4). For example, to represent the changes in the absolute and relative changes in mouse line *Dlg4*, we would need the additional concept “relative increase in lean body mass after body weight adjustment”. However, pre-composing concepts for relative phenotype changes would mean that for each phenotype that is influenced by one or multiple confounders (e.g. body size or length), multiple concepts for each unique phenotype-confounder relationship would need to be added (abnormal/increased/decreased). This would lead to a vast increase in the number of terms (i.e. term explosion) that need to be added and maintained within MP, which would be untenable. This may also be confusing for the user community of curators and annotators as the number and complexity of terms exposed for search and/or annotation grows.

### Tagging pre-composed terms

An adaption to the pre-composed term is to associate an attribute to the annotation by addition of free text tags. This is equivalent to the current implementation used in literature curation at MGI. For example, a gene could possess an annotation “increased lean body mass”, with an annotation or ‘tag’ on this annotation detailing if any/which confounder has been used for adjustment, e.g. “after adjusting for body weight”. However, as the tags are not standardised this may result in non-comparable annotations of genes and an increase in curatorial workload. Furthermore, informatics tools are not capable of interpreting tags of gene annotations and may lead to erroneous presumptions (in the case of a relative change after confounder adjustment that would not be reported with absolute changes only; row 3, Table 1).

In order to disseminate relative phenotype changes to the broader community using tagged pre-composed phenotype ontology annotations, existing gene-annotation databases need to be able to store this additional data and expose this for query. This may require not only changes to the database itself, but also to web interfaces as well as services for data download, in addition to strategies for handling legacy data.

### Standardised qualifiers of pre-composed term

A refinement to the preceding method, is to add standardised qualifiers to the genotype-phenotype annotation. One ontology that can be used to represent these standardised qualifiers is the Phenotype And Trait Ontology (PATO) [18, 19]. The difference between this solution and the previous is that the free text tag is replaced with an ontology term. This suggestion is similar to how sexually dimorphic associations are currently treated. For example, *Kcne2* knockout mice have a number of abnormalities that are specific to the male mice and this is captured as a MP term with associated sex classification tag [20]. The advantage of this solution is that the variability that may occur with free-text tags is reduced to a defined set of ontology concepts. However, following this solution would need an agreed set of ontologies used for the annotation of relative changes and possibly extension to these to account for all possible confounders.

Similar to the latter approach, third parties such as MGI can then choose to add these additional annotations to their data storage to hold the information for relative phenotype changes. This may mean that database schemes as well as provision and distribution methods need to be adapted to handle the additional data and be able to distinguish between absolute and relative phenotype changes. If these changes were to be integrated in existing databases, ways of handling legacy data need to be taken into consideration.

### Post-composed phenotypes

An alternative to pre-composed phenotype annotations is the use of post-composed phenotypes. One method to post-composed phenotypes are entity-quality statements [18, 19], where the phenotype is broken down into an affected entity and a quality describing the entity further, e.g. “increased body weight” (MP:0001260) would be broken down into the entity “multicellular organism” (UBERON:0000468, UBERON is a species-agnostic anatomy ontology) [21] and the quality “increased weight” (PATO:0000582). The following example illustrates how a post-composed ontology-representation could be used to represent a relative phenotype change:

- Entity 1: lean body mass
- Quality: relative to
- Entity 2: body weight
- Qualifier: increased

Applying a post-composed representation to confounder-adjusted phenotypes may lead to multiple sets of annotations to the same set of data as it still needs to be created for each confounder. Where required (e.g. Table 1, row 4), the absolute phenotype change could then be added as it has been done so far with MP annotations or if desired, uniformly with post-composed phenotype annotations.

### Representation of confounder association with RDF triple representation

The ‘**Standardised qualifiers of pre**-**composed terms**’ approach could be formally represented with the Resource Description Framework (RDF) triple model [22]. In an RDF triple, the annotation conforms to the format of < subject, predicate, object>. In our scenarios this would be an MP term as the subject which would be related to the confounder body weight (the object) via the relationship specified as the “relative to” (the predicate). The triple representation is only needed in the annotation arising from including the potential confounders as covariates in the analysis and is a natural extension of the preceding approach ‘**Post composed phenotypes**’.

There are multiple advantages of using RDF models. The first advantage arises from the graphical nature of ontologies in which the inter-relationships of multiple tiers are captured with a graph schema. In an ontology, a class can have multiple parents leading to the inheritance of qualities from different parents, which can be well and efficiently defined within RDF models. The alternative of storing this information is to use a Relational Database Management Systems (RDBMS). In RDBMS, a table scheme is used which faces the computational challenges of multiple joins when querying across many tables and is therefore less scalable. The second advantage is that RDF is a well-established community standard recommended by the World Wide Web Consortium (W3C) [22] and is readily extendible. For example, the same MP term can be associated to other confounders (e.g. body length) using the same predicate. This common structure will lead to a robust data model which will improve efficiency when searching for information. The Ontology for Biomedical AssociatioN (OBAN) is an example of an RDF implementation and has been successfully exploited to represent disease-phenotype associations [23] (Extended version will be published within this special issue) [24].

RDF triples can be stored within relational as well as graphical databases and data queries are performed with the SPARQL query language [25]. In consequence, one would need to understand the technology and the query language to work with the data effectively, through provision of a (non-SPARQL) Application Program Interface (API) would address this for accessing the data.

### Conclusions and future perspectives

In gene-phenotype studies, we have identified challenges with reporting phenotypes after adjusting for body weight using currently available semantic data representation frameworks. Weight is a complex confounder, as it cannot be controlled within the experiment and causality cannot be determined. However, analysing the data with and without body weight returns a richer understanding of the phenotypic abnormality. With interest growing in the impact of body weight on phenotypes and the scale of projects being conducted by high throughput phenotyping consortiums, being able to disseminate annotated phenotype data has become an important issue. We have demonstrated that the impact of including weight as a confounder in the analysis has significant impact on the annotations returned. While this example focuses on the description of mouse phenotypes, we perceive that this is a general problem with accessing phenotypes in all mammals including humans. The current solution implemented with mouse data has arisen from adapting the mechanisms developed for curating literature to a high throughput scenario and use of the ontology for analyses.

We coordinated our efforts with Medical Research Council (MRC) Harwell and MGI in discussions on refining annotation in high throughput phenotyping studies, where MRC Harwell focused on aging studies and how to manage time course studies [10]. The issues were determined to be distinct, as the interpretation is more complex when considering body weight as a confounder. The complexity arises as we cannot determine causality, rather we are annotating the outcome of the statistical analyses.

In the process of this study, we were able to identify several possible solutions (see ‘**Challenges applying existing solutions**’) that could help with applying confounder-relevant information to gene-phenotype associations. These options have been limited to what we believe have the lowest modification requirements on existing dissemination pipelines, such as those maintained by MGI. However, each of these outlined options have to be assessed now in the broader community to arrive at a conclusion what is the best to pursue.

In future work, we aim to not only communicate with the broader community to find the most suitable solution, but also to assess the impact for other potential confounders not just body weight. These additional confounders will then be verified with what has been determined as the best solution to see that it can scale with the demands of the different confounders.

While we have assessed in this study the impact of confounders of gene-phenotype associations in mouse, this is highly likely to be equally relevant in other mammalian model organisms (e.g. rat). However, we identified practical solutions based on the mouse annotation-dissemination pathways and these might not be the optimal for other model organisms. The discussions within this manuscript are a good starting point for managing confounder in their community.

## Additional file

Additional file 1:
**Supplementary Methods.** (DOCX 21 kb)

## Abbreviations

A1: analysis pipeline 1
A2: analysis pipeline 2
API: application program interface
BW: body weight
IMPC: international mouse phenotyping consortium
IMPReSS: international mouse phenotyping resource of standardised screens
MGI: mouse genome informatics database
MGP: mouse genetics project
MP: mammalian phenotype ontology
MRC: medical research council
OBAN: ontology for biomedical association
PATO: phenotype and trait ontology
RDBMS: relational database management systems
RDF: resource description framework
WTSI: wellcome trust sanger institute

## References

[1] Brown SD, Moore MW (2012). Towards an encyclopaedia of mammalian gene function: the International Mouse Phenotyping Consortium. Dis Model Mech.

[2] IMPReSS: International Mouse Phenotyping Resource of Standardised Screens. http://www.mousephenotype.org/impress.

[3] Kurbatova N, Mason JC, Morgan H, Meehan TF, Karp NA (2015). PhenStat: A Tool Kit for Standardized Analysis of High Throughput Phenotypic Data. PLoS One.

[4] Smith CL, Goldsmith C-AW, Eppig JT (2004). The Mammalian Phenotype Ontology as a tool for annotating, analyzing and comparing phenotypic information. Genome Biol.

[5] Hoehndorf R, Hardy NW, Osumi-Sutherland D, Tweedie S, Schofield PN, Gkoutos GV. Systematic Analysis of Experimental Phenotype Data Reveals Gene Functions. PLoS One. 2013;8 (4). doi: 10.1371/journal.pone.0060847 10.1371/journal.pone.0060847 PMC3628905 23626672

[6] Washington NL, Haendel MA, Mungall CJ, Ashburner M, Westerfield M, Lewis SE (2009). Linking human diseases to animal models using ontology-based phenotype annotation. PLoS Biol.

[7] Smedley D, Oellrich A, Kohler S, Ruef B, Westerfield M, Robinson P et al. PhenoDigm: analyzing curated annotations to associate animal models with human diseases. Database. 2013 Jan 1; 2013:bat025 doi: 10.1093/database/bat025. 10.1093/database/bat025 PMC3649640 23660285

[8] Karp NA, Segonds-Pichon A, Gerdin AK, Ramirez-Solis R, White JK (2012). The fallacy of ratio correction to address confounding factors. Lab Anim.

[9] Eppig JT, Blake JA, Bult CJ, Kadin JA, Richardson JE, Mouse Genome Database G. The Mouse Genome Database (MGD): comprehensive resource for genetics and genomics of the laboratory mouse. Nucleic Acids Research. 2012; Jan1;40 (D1):D881-D886. doi:10.1093/nar/gkr974. 10.1093/nar/gkr974 PMC3245042 22075990

[10] Greenaway S, Blake A, Retha A, O’Leary J, Goldsworthy M, Bowl MR (2015). Automatically annotating temporal data from a phenotype-driven mutagenesis screen. Proceedings of Phenotyping Day at ISMB, Dublin, Ireland.

[11] Reed DR, Lawler MP, Tordoff MG (2008). Reduced body weight is a common effect of gene knockout in mice. BMC Genet.

[12] Karp NA, Melvin D, Mott RF, Project SMG. Robust and Sensitive Analysis of Mouse Knockout Phenotypes. PLoS One. 2012;7 (12). doi:10.1371/journal.pone.0052410. 10.1371/journal.pone.0052410 PMC3530558 23300663

[13] Tschop MH, Speakman JR, Arch JR, Auwerx J, Bruning JC, Chan L (2011). A guide to analysis of mouse energy metabolism. Nat Methods.

[14] Anika O, Meehan T, Parkinson H, Sarntivijai S, White JK, N.A. K. Supporting data: Reporting phenotypes in model organisms when considering body size as a potential confounder. 2015. https://zenodo.org/record/32082?ln=en#.ViYKnE2FOiM. 10.1186/s13326-016-0050-8 PMC4748495 26865945

[15] Karp NA, Speak AO, White JK, Adams DJ, Hrabe de Angelis M, Herault Y et al. Impact of temporal variation on design and analysis of mouse knockout phenotyping studies. PLoS One. 2014;9 (10):e111239. doi:10.1371/journal.pone.0111239. 10.1371/journal.pone.0111239 PMC4208881 25343444

[16] White JK, Gerdin AK, Karp NA, Ryder E, Buljan M, Bussell JN (2013). Genome-wide Generation and Systematic Phenotyping of Knockout Mice Reveals New Roles for Many Genes. Cell.

[17] MGI. Dmxl2tm1a (EUCOMM) Wtsi Phenotyping Page. http://www.informatics.jax.org/allele/MGI:4432280.

[18] Gkoutos GV, Mungall C, Dolken S, Ashburner M, Lewis S, Hancock J et al. Entity/Quality-Based Logical Definitions for the Human Skeletal Phenome using PATO. In Engineering In Medicine and Biology Society. 2009:7069–72. doi:10.1109/Iembs.2009.5333362. 10.1109/IEMBS.2009.5333362 PMC3398700 19964203

[19] Mungall CJ, Gkoutos GV, Smith CL, Haendel MA, Lewis SE, Ashburner M. Integrating phenotype ontologies across multiple species. Genome Biology. 2010;11 (1). doi:10.1186/Gb-2010-11-1-R2. 10.1186/gb-2010-11-1-r2 PMC2847714 20064205

[20] MGI. Kcne2tm1a (EUCOMM) Wtsi Phenotyping Page. http://www.informatics.jax.org/allele/MGI:4431909.

[21] Haendel MA, Balhoff JP, Bastian FB, Blackburn DC, Blake JA, Bradford Y et al. Unification of multi-species vertebrate anatomy ontologies for comparative biology in Uberon. Journal of Biomedical Semantics. 2014; 5 (21). doi:10.1186/2041-1480-5-21. 10.1186/2041-1480-5-21 PMC4089931 25009735

[22] Lassila O, Swick RR (1999). Resource Description Framework (RDF) model and syntax specification.

[23] Sarntivijai S, Vasant D, Saunders G, Bento P, Gonzalez D, Betts J (2015). Linking rare and common disease: mapping clinical disease-phenotypes to ontologies in therapeutic target validation. Proceedings of Phenotyping Day at ISMB, Dublin, Ireland.

[24] Brush MH, Mungall C, Washington NL, Haendel M, What’s in a Genotype? (2013). An Ontological Characterization for Integration of Genetic Variation Data. Proceedings of lnternational Conference on Biomedical Ontology.

[25] Feigenbaum L, Williams GT, Clark KG, Torres E (2013). SPARQL 1.1 protocol. Recommendation, W3C.

[26] Koscielny G, Yaikhom G, Iyer V, Meehan TF, Morgan H, Atienza-Herrero J (2013). The International Mouse Phenotyping Consortium Web Portal, a unified point of access for knockout mice and related phenotyping data. Nucleic Acids Res.

