# Supplementary material for: Reporting phenotypes in mouse models when considering body size as a potential confounder
Source: J Biomed Semantics. 2016 Feb 9;7:2. doi: 10.1186/s13326-016-0050-8 (PMC4748495; doi:10.1186/s13326-016-0050-8)
Supplement: Additional file 1: — Supplementary Methods. (DOCX 21 kb) [file 13326_2016_50_MOESM1_ESM.docx]

**Supplementary Methods**

**Ethical approval:** The care and use of mice in the WTSI study was carried out in accordance with UK Home Office regulations, UK Animals (Scientific Procedures) Act of 1986 under two UK Home Office licences which approved this work (80/2076 and 80/2485) which were reviewed regularly by the WTSI Ethical Review Committee. All efforts were made to minimize suffering by considerate housing and husbandry. Animal welfare was assessed routinely for all mice involved. Adult mice were killed by terminal anaesthesia followed by exsanguination and either cervical dislocation or removal of the heart.

**Mouse generation:** Mice carrying knockout first conditional-ready alleles were generated as described previously [1]. Data for all lines are available from the [IMPC web portal](http://www.knockoutmouse.org/) [2]**,** excerpts are presented here.

**Genotyping and allele quality control:** Embryonic stem cell quality control was performed as described [3]. Furthermore, molecular characterisation of mutant mouse strains was performed as described previously [4]. Upon completion of phenotyping, genotyping was repeated and data were only accepted from mice for which the second genotype was concordant with the P14 genotype.

**Housing and husbandry:** Mice were maintained in a specific pathogen free unit on a 12hr light: 12hr dark cycle with lights off at 7:30pm and no twilight period. The ambient temperature was 21 ± 2^o^C and the humidity was 55 ± 10%. Mice were housed for phenotyping using a stocking density of 3-5 mice per cage (overall dimensions of caging: (L x W x H) 365 x 207 x 140mm, floor area 530cm^2^) in individually ventilated caging (Tecniplast Seal Safe1284L) receiving 60 air changes per hour. In addition to Aspen bedding substrate, standard environmental enrichment of two nestlets, a cardboard Fun Tunnel and three wooden chew blocks was provided. Mice were given water and diet *ad libitum,* unless otherwise stated. Mice on the pipelines named “Pipeline 2” and “MGP Select” were maintained on Mouse Breeders Diet (LAb Diets, 5021-3) throughout their lifetime. At 4 weeks of age, mice on the pipelines named “Pipeline 1” and “Mouse GP”, were transferred from Mouse Breeders Diet (Lab Diets, 5021-3) to a high fat (21.4% fat by crude content; 42% calories provided by fat) dietary challenge (Special Diet Services, Western RD 829100). For the *Dlg4*^tm1e^*^(EUCOMM)Wtsi^* example, the data were collected from pipeline MGP Select, whilst the *Akt2*^tm1e(KOMP)Wtsi^ data were collected from Pipeline 1.

**Phenotyping:** The analysis uses data taken from high-throughput phenotyping analysis, which is based on a pipeline concept where a mouse is characterised by a series of standardised and validated set of tests underpinned by standard operating procedures. The phenotyping tests chosen cover a variety of disease-related and biological systems, including the metabolic, cardiovascular, bone, neurological and behavioural, sensory and haematological systems and clinical chemistry. The data were obtained as detailed in [1] following the standard operator procedures at IMPReSS [5]. Factors thought to affect the variables were standardised as far as possible. Where standardisation was not possible, steps were taken to reduce potential bias. For example, at WTSI a common strategy used to minimise the impact of different people completing the experiment (“minimised operator”) is defined in the [Mouse Experimental Design Ontology](http://bioportal.bioontology.org/ontologies/MEDO) as “The process by which steps are taken to minimise the potential differences in the effector by training and monitoring of operator.” [6]. Further detailed experimental design information (e.g. methods used for each SOP) were captured with an ontology as detailed in Karp *et al* [7] and is available from the IMPC portal at the ARRIVE webpage [8].

**Experimental design:** Phenotyping data is collected at regular intervals on age-matched control (wildtype) mice of equivalent genetic backgrounds. Cohorts of at least seven homozygote mice of each sex per knockout line were generated. If no homozygotes were obtained from 28 or more offspring of heterozygote intercrosses, the line was deemed non-viable. Similarly, if less than 13% of the pups resulting from intercrossing were homozygous, the line was judged as being subviable. In such circumstances, heterozygote mice were committed to the phenotyping pipelines.

The random allocation of mice to experimental group (wildtype versus knockout) was driven by Mendelian Inheritance. Reflecting the high-throughput nature of the phenotyping pipeline, blinding to the identity of knockout lines during phenotyping was not employed as the cage cards include genotype information, though with a high throughput environment without defined hypothesis the potential bias is minimised. The individual mouse was considered the experimental unit within the studies. Further detailed experimental design information (e.g. exact definition of a control animal) for WTSI is captured by a standardised ontology as detailed in Karp *et al* [7] and is available from the IMPC portal (http://www.mousephenotype.org/about-impc/arrive-guidelines).

**Data Quality Control (QC):** Pre-set reasons are established for QC failures (e.g. insufficient samples) and detailed within IMPRESS providing standardised options as agreed by area experts as to when data can be discarded. Data can only be QC failed from the dataset if clear technical reasons can be found for a measurement being an outlier. Reasons are provided and tracked within the database.

**Datasets:** The Wellcome Trust Sanger Institute (WTSI) dataset consists of data from 831 knockout lines for a number of different phenotypic characteristics. Within the dataset there are 85086 wildtype-knockout subsets of wildtype and knockout data which have been associated for a variable of interest allowing for statistical comparisons. Control data was selected to have been collected on the same pipeline, same genetic background, same standard operating procedure, and spanning the same time period over which the knockout mice were studied. If the knockout mice for a line were studied for a time period shorter than six months, then the time window was increased to ensure a minimum of six months’ worth of control data was selected centred over the period over which knockout data were collected.

**Statistical and bioinformatics analysis:** An iterative top down mixed modelling strategy was performed as described in [9] using PhenStat an R package version 2.0.1 [10] freely available from Bioconductor [11] with Eq. 1 as the starting model for Analysis Pipeline 1 (A1) and Eq. 2 for Analysis Pipeline 2 (A2).

dependent Variable ~ Genotype + Sex + Genotype*Sex + (1|Batch) [Eq. 1]

dependent Variable ~ Genotype + Sex + Genotype*Sex + Weight + (1|Batch) [Eq. 2]

The genotype effect was classed as statistically significant if the genotype contribution test returned a p value below the IMPC significance threshold of 0.0001[7].

When a covariate is included in a regression analysis, the statistical model adjusts for the confounding variable by estimating the linear relationship between the confounding variable and the variable of interest and as such makes two assumptions. Firstly, that there is a linear relationship between the confounding variable and the variable of interest and secondly the assumption that a common linear relationship exists (homogeneity of regression slope). These assumptions can be assessed by plotting the confounding variable (body weight) against the outcome (phenotypic measure) and comparing the gradients on regression lines fitted for each experimental condition (genotype). In a large scale automated analysis, visual inspections of graphs are not possible. Within PhenStat, a model optimisation strategy has been implemented and therefore if weight as a covariate is not found to be a significant source of variation it is dropped from the modelling process. This statistical method has been studied through simulations and resampling studies [12] and found to be robust and reliable with a multi-batch workflow, where the knockout mice are split into multiple phenotyping batches.

**Data Access:** Data used within this manuscript is freely available from the IMPC project portal [2]. Data can be downloaded via the Experimental Data REST API and instructions for using this application program interface are available at associated GitHub page [13]. The statistical analysis output files and associated script files are available at Zenodo [14].

**References:**

1. White JK, Gerdin AK, Karp NA, Ryder E, Buljan M, Bussell JN et al. Genome-wide Generation and Systematic Phenotyping of Knockout Mice Reveals New Roles for Many Genes. Cell. 2013;154(2):452-64. doi:10.1016/j.cell.2013.06.022.

2. IMPC. IMPC project portal. [www.mousephenotype.org](http://www.mousephenotype.org/).

3. Skarnes WC, Rosen B, West AP, Koutsourakis M, Bushell W, Iyer V et al. A conditional knockout resource for the genome-wide study of mouse gene function. Nature. 2011;474(7351):337-U61. doi:10.1038/Nature10163.

4. Ryder E, Gleeson D, Sethi D, Vyas S, Miklejewska E, Dalvi P et al. Molecular characterization of mutant mouse strains generated from the EUCOMM/KOMP-CSD ES cell resource. Mammalian Genome. 2013;24(7-8):286-94. doi:10.1007/s00335-013-9467-x.

5. IMPReSS: International Mouse Phenotyping Resource of Standardised Screens.<http://www.mousephenotype.org/impress>.

6. Parkinson H, N.A. K, IMPC. Mouse Experimental Design Ontology. 2012.<http://bioportal.bioontology.org/ontologies/MEDO/?p=summary>.

7. Karp NA, Meehan TF, Morgan H, Mason JC, Blake A, Kurbatova N et al. Applying the ARRIVE Guidelines to an In Vivo Database. PLoS Biology. 2015;13(5). doi:ARTN e1002151

10.1371/journal.pbio.1002151.

8. IMPC. ARRIVE Guidelines and the IMPC. 2014. https://[www.mousephenotype.org/about-impc/arrive-guidelines](http://www.mousephenotype.org/about-impc/arrive-guidelines).

9. Karp NA, Melvin D, Mott RF, Project SMG. Robust and Sensitive Analysis of Mouse Knockout Phenotypes. PLoS One. 2012;7(12). doi:10.1371/journal.pone.0052410.

10. Kurbatova N, Mason JC, Morgan H, Meehan TF, Karp NA. PhenStat: A Tool Kit for Standardized Analysis of High Throughput Phenotypic Data. PloS One. 2015;10(7):e0131274. doi:10.1371/journal.pone.0131274.

11. Gentleman RC, Carey VJ, Bates DM, Bolstad B, Dettling M, Dudoit S et al. Bioconductor: open software development for computational biology and bioinformatics. Genome Biology. 2004;5(10):R80. doi:10.1186/gb-2004-5-10-r80

12. Karp NA, Speak AO, White JK, Adams DJ, Hrabe de Angelis M, Herault Y et al. Impact of temporal variation on design and analysis of mouse knockout phenotyping studies. PLoS One. 2014;9(10):e111239. doi:10.1371/journal.pone.0111239.

13. Mason J. Experimental data REST API. 2014. https://github.com/mpi2/PhenotypeArchive/wiki/Experimental-data-REST-API.

14. Anika O, Meehan T, Parkinson H, Sarntivijai S, White JK, N.A. K. Supporting data: Reporting phenotypes in model organisms when considering body size as a potential confounder. 2015. https://zenodo.org/record/32082?ln=en#.ViYKnE2FOiM.
